# Apelin-13 and Asprosin in Adolescents with Anorexia Nervosa and Their Association with Psychometric and Metabolic Variables

**DOI:** 10.3390/nu14194022

**Published:** 2022-09-28

**Authors:** Katarzyna Jowik, Monika Dmitrzak-Węglarz, Natalia Pytlińska, Anna Jasińska-Mikołajczyk, Agnieszka Słopień, Marta Tyszkiewicz-Nwafor

**Affiliations:** 1 Department of Child and Adolescent Psychiatry, Poznan University of Medical Sciences, 61-701 Poznan, Poland; 2 Department of Psychiatric Genetics, Poznan University of Medical Sciences, 61-701 Poznan, Poland; 3 Department of Adult Psychiatry, Poznan University of Medical Sciences, 61-701 Poznan, Poland

**Keywords:** anorexia nervosa, apelin, asposin

## Abstract

Anorexia nervosa (AN) is a widespread, metabo-psychiatric disorder with high relapse rates, comorbidity, and mortality. Many regulatory proteins and neurohormones studied to date play essential roles in the etiopathogenesis of eating disorders and the maintenance of psychopathological symptoms. Nevertheless, the regulatory and pathophysiological mechanisms of AN are still poorly understood. In the presented study, the plasma levels of apelin-13 (APE-13) and asprosin (ASP), as well as carbohydrate metabolism parameters and psychometric parameters, were evaluated in low-weight adolescent female patients with AN (AN1), after partial weight normalization (AN2) and in an age-matched healthy control group (CG) were evaluated. APE-13 levels were higher in the AN1 group than in the post-realimentation and the CG group. APE-13 levels were independent of insulin and glucose levels. Plasma ASP levels increased with increasing body weight in patients with AN, correlating with the severity of eating disorder symptoms in emaciation. The presented data suggest that APE-13 and ASP may be AN’s biomarkers-regulation of eating behavior by APE-13 and ASP, the close relationship between them and emotional behavior, and changes in neurohormone levels in patients with eating and affective disorders seem to support these hypotheses. Moreover, their plasma levels seem to be related to the severity of psychopathological symptoms of eating disorders.

## 1. Introduction

Anorexia nervosa (AN) is a widespread, still poorly understood metabo-psychiatric disorder with high relapse rates, comorbidity, and mortality [1,2,3]. It is a multifactorial disorder with a strong genetic component. The psychobiological and sociocultural factors may influence AN’s development, as may abnormalities in regulatory peptides [4,5]. Complex regulatory mechanisms involving homeostatic and nonhomeostatic processes control eating behaviors and emotions. Regulatory proteins play an essential role in monitoring food intake by influencing hypothalamic control of feeding. In addition, they can also affect processes connected with food intake through their receptors in the cortico-limbic system. Emotions, motivation, physical activity, and cognitive deficits in attention, executive function, and mentalization are crucial for regulating food intake and the AN’s etiology [6,7]. 

Asprosin (ASP), encoded by two exons of the Fibrillin 1 (FBN1) gene, was discovered in 2016 as a glucogenic protein and adipokine. Recent studies have shown that it plays an essential and complex role in metabolism and metabolic diseases, acting at both central and peripheral levels and being involved in appetite regulation, insulin resistance, glucose metabolism, and cell apoptosis [8,9,10]. ASP can cross the blood–brain barrier and function as an appetite regulator by stimulating orexigenic agouti-related neuropeptide (AgRP) neurons via a cAMP-dependent signaling pathway and by inhibiting anorexigenic proopiomelanocortin (POMC) neurons in a GABA-dependent manner [9,11,12]. The ASP properties emerging from Duershmidt’s mouse study indicate the glucogenic and orexigenic nature of this protein and its high sensitivity to the body’s energy status. The central (neuronal) action of ASP may affect its peripheral (hepatic) activity or vice versa. Nevertheless, the pathological increase in ASP in insulin resistance and obesity and the observed efficacy in immune neutralization using antibodies against ASP in mice suggest that loss of ASP function may serve as a potentially unique therapeutic avenue for treating metabolic diseases [9]. Additionally, ASP may also raise blood glucose levels during fasting. According to Jean Mayer’s hypothesis, changes in blood glucose levels may regulate satiety and eating behavior [13,14]. A study evaluating plasma ASP levels in cancer patients with anorexia and cachexia showed that its levels did not differ significantly between patients with or without cachexia, but patients with anorexia had significantly lower ASP levels compared to patients without anorexia [15]. The authors noted that the exact mechanism involved in the effect of ASP on appetite control in cancer patients remains a topic for future research. It seems necessary to identify the possible use of this agent as a therapeutic option for anorexia, with a diverse primary pathogenesis. Studies have also shown that lower postprandial glucose levels in patients with AN may be related to delayed gastric distension [16]. Although gastric emptying improves when refeeding AN patients, postprandial glucose levels are still low [17]. 

Apelin (APE) is an adipose tissue-derived peptide widely distributed in the CNS and peripheral tissues that control feeding behavior, energy homeostasis, and neuroendocrine function [18]. Existing evidence for the importance of APE, especially in children, and its association with obesity and cardiometabolic risk factors remains unclear, and the results are inconsistent. Some studies have shown increased levels in obese compared to normal-weight subjects [19,20,21,22]. In contrast, other studies have found no significant changes or even lower levels in obese children than in healthy ones [23,24,25,26]. Several different isoforms of APE have been identified, of which APE-36 is supposed to be the dominant one. At the same time, the biological activity of APE is inversely proportional to the length of the peptide; thus, APE-12 and APE-13 are the most active isoforms. The plasma levels of APE-13, a major neuroprotective peptide, are the highest. As an endogenous ligand of angiotensin type 1 receptor domain-related protein (APJ), the APE-13 system is involved in several physiological and pathological processes, such as vasculopathy, energy metabolism, and maintenance of humoral homeostasis [27,28]. The association of APE with the APE-12 isoform and APE-36 has been studied in the context of bone metabolism in AN patients, showing a negative effect on the balance of the osteoprotegerin/activator of nuclear factor κB ligand receptor (OPG/RANKL) system while negatively affecting the bone remodeling process in AN patients [29]. Experimental studies have demonstrated the anti-inflammatory and neuroprotective effects of APE-13, inhibition of autophagy, and regulation of apoptosis in neurodegenerative diseases [30,31].

Most previous studies have examined the role of ASP and APE-13 in adult AN patients. To the best of our knowledge, no studies of ASP in juvenile AN patients have been designed to date, and studies on APE are few and involve protein subtypes other than APE-13. The above factors have become the focus of studies mainly on adults and obese individuals. It is not clear whether the regulation of food intake in developing adolescents is the same as in adults. Nevertheless, the evaluation of the adolescent population may allow us to observe regulatory mechanisms that counteract neuroplastic and metabolic changes caused by long disease duration. Given the demonstrated positive correlations between plasma APE-13 levels and BMI and the marked fat deficit in AN, we hypothesized that the spectrum of APE-13 biological activity is associated with the negative consequences of long-term starvation. Therefore, we hypothesized that the plasma levels of APE-13 in patients with AN are significantly lower than in healthy subjects. Furthermore, we hypothesized that ASP would change with weight gain, as well as parameters of carbohydrate metabolism, such as fasting glucose, insulin, and HOMA index. Therefore, the present study aimed to investigate the plasma levels of ASP and APE-13 in malnourished (AN1) and partially cured (AN2) adolescent patients with AN. Correlations between protein levels and several dimensions of AN symptomatology, such as eating disorder, depressive, and obsessive compulsive symptoms, were investigated. 

## 2. Materials and Methods

Sixty-four patients aged 11–18 years admitted to the Department of Child and Adolescent Psychiatry in the acute phase of AN participated in the study. The methodology of this study is similar to other studies previously published by our team [32,33,34]. Informed written consent was obtained from the participants and their legal guardians. The diagnosis of restrictive AN was made according to ICD-10 criteria, following a semistructured interview by a child and adolescent psychiatrist. A physical examination and basic biochemical tests were performed. Exclusion criteria included physical illnesses and laboratory abnormalities not attributable to prolonged food restriction, as well as psychiatric comorbidities. Between the 1st and 3rd days of admission, patients with AN (AN1) underwent psychometric evaluation, height and weight assessment, and 15 mL of blood was drawn. The same procedures were repeated at a second time point about 11.2 ± 2.3 weeks later, after partial normalization of body weight on the day of discharge (AN2). Forty-four patients who partially regained weight completed the study. Twenty patients who failed to gain weight or were discharged against medical advice were excluded. The control group (CG) consisted of twenty-nine healthy, normal-weight girls with no history of psychiatric disorders, recruited from among the students of a local school. They underwent physical and psychiatric examination, anthropometric and psychometric evaluation, and blood analysis. BMI was calculated as the ratio of body weight (kg) to height (m^2^), and percent ideal body weight (%IBW) was calculated as the ratio of actual to ideal body weight (IBW) × 100%, where IBW (kg) = [height (cm) − 100] − {[height (cm) − 150]/2} according to the Lorentz formula. All measurements were taken in standing, fasting women. The Eating Attitudes Test (EAT-26), Beck Depression Inventory (BDI) Hamilton Depression Scale (HAMD) and Yale–Brown Obsessive Compulsive Scale (CYBOCS), used in clinical practice and research in Polish adolescent girls, were used to assess eating disorder symptoms, depression, obsessions and compulsions. Patients were included in a nutritional rehabilitation program. Daily caloric intake was 2000–2500 kcal and was gradually increased to 3500–4000 kcal depending on weight gain (1.0–1.5 kg per week). Patients with acute symptoms, such as severe agitation, anxiety or insomnia, received ad hoc medication, usually hydroxyzine, benzodiazepines or small doses of atypical antipsychotics. 

The Bioethics Committee approved the University of Medical Sciences’ study protocol (1029/13). All procedures were carried out in accordance with the 1964 Declaration of Helsinki. 

### 2.1. Biochemical Analysis

Venous blood was collected on morning admission (8:00–9:00 a.m.) from fasting (12 h after the last meal) AN, and CG subjects. Serum was immediately separated from the blood by centrifugation at 1000× *g* for 15 min at 4 °C, aliquoted into Eppendorf tubes, frozen at −70 °C, and assayed afterwards. Quantitative ASP and APE-13 tests were performed using a commercial one enzyme-linked immunosorbent assay (Human ASPRO/Asprosin ELISA Kit Asprosin, cat. No. E15190h, and Human APLN13/Apelin-13 ELISA Kit, cat. No. E1887h), produced by EIAab, following the manufacturer’s instructions. Immunoenzymatic test for APE-13 was specific only for isoform 13 according to the manufacturer. The measurement range of the kit was 1.56–100 ng/mL for ASP and 78–5000 pg/mL for APE-13. The minimum detectable dose of general ASP is typically less than 0.808 ng/mL CVs% inter-assay < 9.8% and intra-assay < 6.5% and for APE-13 less than 33 pg/Ml, CVs% inter-assay < 6.5% and intra-assay < 4.0%. Optical density was read via a spectrophotometric plate reader (Biochrom Asys UVM 340 Microplate Reader) at a wavelength of 450 ± 10 nm. Every assay was performed twice, and the mean value of the two assays was used for statistical evaluation. A four-parameter algorithm (four-parameter logistic) was used to assay the concentration in the tested samples.

### 2.2. Statistics

Analyses were conducted in statistical software R, version 4.1.3. All variables measured in a study were quantitative ones. The normality of distributions was checked with Shapiro–Wilk’s test and with skewness value. Dependent samples (AN1 and AN2 groups) were compared using Student’s *t*-test for dependent groups (paired *t*-test) or Wilcoxon’s test. Independent samples (AN groups and control group) were compared with Student’s *t*-test for independent groups or Mann–Whitney’s *U* test. The AN1 and AN2 groups represent a group of the same patients tested at different time points, which precluded the use of an ANOVA test. Spearman’s rho coefficient was used for correlation analyses as it is resistant to outliers and non-normal distributions. Scatter plots were made to show the direction of significant relationships between APE-13 or ASP and other selected variables. Regression models were not built as the assumption about the linear relationship between predictors and the dependent variable was not met. Statistical analysis was based on a significance level of 0.05.

## 3. Results

### 3.1. Demographic Data and Clinical Assessments

Table 1 compares demographic and clinical data between the AN1, AN2, and the CG. The mean age of onset in AN patients was 15.5 years. Subjects from the experimental group (AN1 and AN2) were significantly shorter and had a lower IBW than the CG (*p* = 0.024 for both analyses). Patients with AN in the malnourished state (AN1) had lower body weight, BMI, and %IBW than after partial weight normalization (AN2) and than those from the CG (*p* < 0.001 for all analyses). Patients from the AN2 group also had lower body weight, BMI, and %IBW than subjects from the CG (*p* < 0.010). As presented in Table 2, the BDI, HAMD, CYBOCS and EAT-26 scores were significantly higher among patients from AN1 than AN2 and higher than in the CG (*p* < 0.050 for all analyses). The BDI and HAMD scores were also significantly higher in patients from AN2 group than in the CG (*p* ≤ 0.050 for all significant analyses). 

### 3.2. Asprosin and Apelin-13 Levels

**Figure 1:**
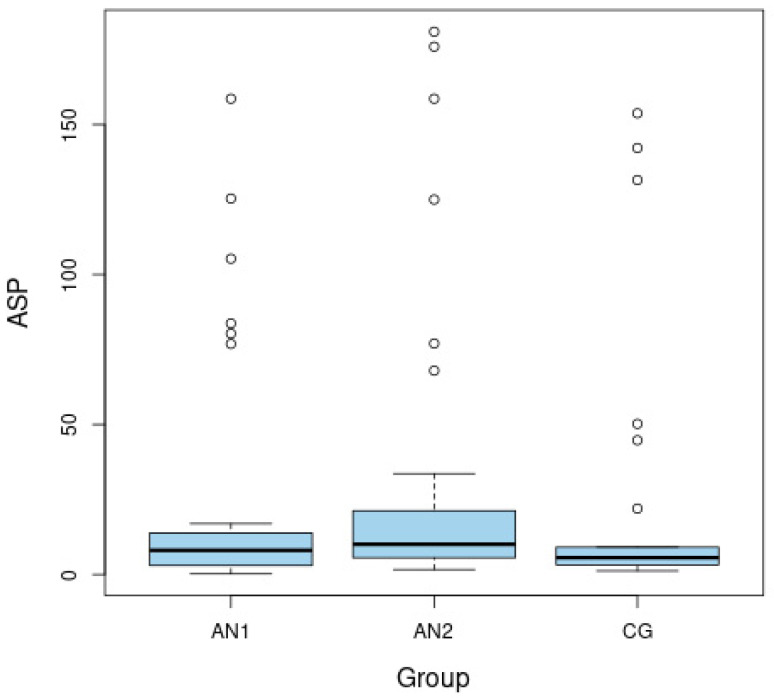
Boxplots of fasting levels of asprosin (ASP) for adolescents with anorexia nervosa in extremely low body weight (AN1), after partial normalization of body weight (AN2), as well as in the healthy control group (CG).

**Figure 2:**
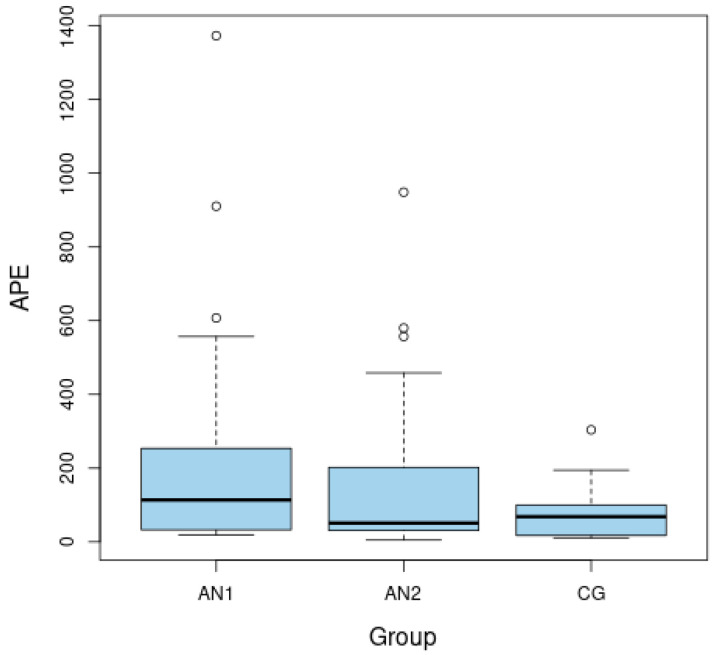
Boxplots of fasting levels of apelin-13 (APE-13) for adolescents with anorexia nervosa in extremely low body weight (AN1), after partial normalization of body weight (AN2), as well as in the healthy control group (CG).

The ASP level was significantly lower among malnourished patients than after weight normalization (*p =* 0.008 for AN1 vs. AN2) (Figure 1). The APE-13 level was higher in the patients from AN1 than from AN2 group and higher than in the CG (*p* = 0.037 for AN1 vs. AN2 and *p* = 0.046 for AN1 vs. CG) (Figure 2). The insulin level and HOMA-IR were significantly lower among patients in the AN1 than in the AN2 group and lower than in the CG (*p* < 0.050 for all analyses). The HOMA-IR level was higher in patients from the AN2 than in subjects from the CG (*p* = 0.026). (Table 1).

### 3.3. Correlations between Asprosin and Apelin-13 and Metabolic Variables in AN Patients

Table 3 shows that ASP levels were significantly negatively correlated with body weight (rho = −0.35; *p* = 0.032), height (rho = −0.32; *p* = 0.0497) and IBW (rho = −0.32; *p* = 0.0497), but were not correlated with BMI (rho = −0.26; *p* = 0.113) and glucose levels (rho = −0.23; *p* = 0.175) in AN1, while in AN2 it correlated negatively with height (rho = −0.50; *p* = 0.001), glucose levels (rho = −0.33; *p* = 0.037), insulin levels (rho = −0.43; *p* = 0.005) and HOMA-IR (rho = −0.49; *p* = 0.002), and positively with BMI (rho = 0.34; *p* = 0.028). It did not correlate with body weight (rho = 0.04; *p* = 0.818). 

The APE-13 level was significantly positively correlated to IBW (rho = 0.43; *p* = 0.008) but only in the AN2. No significant correlation was observed among subjects from the CG.

### 3.4. Relationships between Asprosin and Apelin-13 and Psychometric Variables in AN Patients

As presented in Table 4, among patients in AN1, ASP correlated positively with EAT-26 scores (rho = 0.51; *p* = 0.025), but no correlation was shown for other scales (Table 4.). APE-13 was significantly negative correlated to: BDI (rho = −0.46; *p* = 0.034) and EAT-26 scores (rho = −0.50; *p* = 0.028). After weight normalization, in the AN2 group, ASP levels showed no correlation with psychometric test scores, while APE-13 levels correlated positively with EAT-26 scores (rho = 0.53; *p* = 0.030). Similarly to metabolic results, no significant correlation was observed among subjects from the CG. BDI was only associated with APE among AN1 subjects. The remaining correlations of depressive symptom levels and APE and ASP concentrations were not significant. No correlation was detected between ASP and APE with BDI or HAM when patients were analyzed together without splitting into groups.

## 4. Discussion

Patients with AN and healthy subjects under the age of 18 years participated in the study. They differed in body weight, BMI, %IBW and the severity of the studied psychopathological symptoms. Patients diagnosed with AN presented significantly higher scores on depression scales (BDI and HAMD) than the CG group, which is consistent with reports that up to 70% of patients with AN have comorbid depressive symptoms. Our results confirm that inpatient treatment of AN is associated with reduced depression and eating disorder ed (EAT-26) as reported in previous studies [35,36]. Moreover, AN1 group had higher obsessiveness and compulsivity measured with the CYBOCS scale than the AN2 and the CG group. This has also been reported in Amiato’s study and supports Levinson’s hypothesis of likely common etiological pathways [37,38]. The OCD symptoms occur in 35–44% of patients with AN, and AN occurs in 10% of women with OCD. Interestingly, patients after nutritional rehabilitation did not have statistically different obsessiveness scores than the CG, which is consistent with the results obtained by Kucharska [39]. Concerning eating disorder symptoms per se, there was a significant improvement in the post-treatment group, although their severity remained higher compared to healthy individuals.

The pathogenesis of AN involves several genetic, neurobiological, psychological and socio-cultural factors, and increasing evidence points to an important role in metabolic dysfunctions, including disturbances at the level of glucose, insulin and IGF-1 regulation [40,41,42]. The lower insulin and HOMA-IR levels observed in AN1 than in AN2 and CG probably allow for the involvement of counter-regulatory mechanisms of long-term starvation. Paradoxically, insulin levels are elevated early in the realignment process (AN2), as Bulik also points out while describing the mechanism of reduced IGF-1 levels [5,43]. The acquired growth hormone (GH) resistance observed in patients with AN with low levels of insulin-like growth factor-1 (IGF-1) and changes in the activity of the GH/IGF-I axis reflect an impaired nutritional state and can be considered one of the basic metabolic features of the disease, which is generally restored by nutrition and stable weight gain [44,45]. At the same time, the above changes are only part of the picture of AN as a metabo-psychiatric disease.

Numerous studies have shown that ASP may be a biomarker indicating levels of fat mass and a target for the treatment of metabolic disorders. Until today, it has not been possible to confirm in observational studies a cause-and-effect relationship. Studies of patients with Neonatal Progeroid Syndrome (NPS), caused by mutations in the FBN1 gene, show a deficiency of plasma ASP, resulting in extreme emaciation and fat deficiency. Measured food intake and energy expenditure differed from the healthy control group. Studies in animals and the introduction of mutations in their FBN1 gene allowed us to observe that Fbn1 NPS/+ mice exhibit extreme leanness compared to sex-matched individuals. To ascertain whether ASP stimulates appetite, the researchers administered a dose of recombinant ASP to the rodents, observing that the individuals showed greater food intake over the next 24 h. Hence, we conclude that ASP is essential for regulating and maintaining normal appetite levels. Moreover, its action is likely based on inhibiting anorexigenic POMC + neurons in the arcuate nucleus and activating AgRP + neurons [9,10,46]. 

Our study showed that adolescents in the acute phase of AN (AN1) do not have significantly higher plasma ASP concentrations than controls, which differs from Hu’s results. This may be explained by impaired fasting ASP secretion [10,47]. Given its function as an orexigenic hormone, the increase in circulating ASP in patients with AN is probably due to the starvation response as compensation for the body’s energy balance. 

Intriguingly and confusingly, ASP after nutritional rehabilitation (AN2) was higher than in extremely low body weight. We suspect that fasting is not the sole determinant of ASP regulation. Similar to Hu, we observe that in the acute phase of AN, plasma ASP levels correlated positively with eating disorder psychopathology. 

Although ASP promotes hepatic glucose release, causing hyperglycemia, patients with AN typically have relatively low blood glucose levels due to inadequate energy supply and depletion of glucogenic substrates. Furthermore, some studies show a positive correlation between ASP and fasting glucose levels, others, like ours, do not show a similar relationship [47,48,49,50,51,52]. This may be related to the precise glucose regulation by several hormones, and ASP is only one of them. Evidence for the above thesis may also be provided by the fact that ASP treatment does not affect the levels of glucogenic hormones and plays a complex role in glucose release independent of them. We suspect that the fact that ASP levels do not normalize with increasing body weight may be relevant to the chronicity of some of the disease symptoms.

APE, a peptide derived from adipose tissue, might be one of the leading factors in developing metabolic disorders, but its role remains unclear, and experimental results are inconsistent [53]. In studies of obese patients, plasma APE levels were reduced compared to healthy patients [25,26]. At the same time, in studies of obese children, its level was significantly higher than in non-obese children [23]. These opposite findings can be explained by the differential expression of APE in tissues. In obese and insulin-resistant mice on a high-fat diet, plasma APE-12 levels were unchanged. Moreover, APE gene expression levels were elevated in white adipose tissue and decreased in brown adipose tissue, liver, and kidneys, suggesting that the apelinergic system may be involved in the several different dysfunctions coexisting in obesity-related disease [54]. Additionally, plasma APE levels were significantly reduced in obese patients after weight loss induced by a low-calorie diet. Diet-induced changes in plasma APE levels are directly correlated with diet-induced decreases in insulin and TNF-α levels [55]. 

To the best of our knowledge, this is the first published report of plasma APE-13 levels in adolescent female patients with AN. Results showed that plasma APE-13 concentrations were significantly higher in girls with AN than in CG. The correlations between APE-13 concentrations and eating disorder outcomes in AN2 patients were also reported. AN2 patients showed statistically lower protein levels than AN1 group. The study results conducted by Ziora are opposite, but they examined other APE subunits, namely APE-36 and APE-12 [56]. Moreover, in the Vehapoglu study conducted on a similarly small group of underweight children, APE scores were significantly lower than in normal-weight children [57]. Notably, the subjects were prepubertal, of both sexes, and did not exhibit features of eating disorders as our patients did. We speculate that APE secretion in AN patients is regulated in a complex multifactorial manner, dependent on metabolic and psychopathological factors. 

No similar studies on APE-13 in children and adolescents have been found. We speculate that the differences in the results may be related to the age of the responders, especially since animal studies have shown that APE-13 mRNA expression levels are age-dependent and decrease with age Moreover, future experiments should investigate whether AN’s duration affects the peptide level changes. Of the many APE isoforms, APE-13 is a major neuroprotective peptide and signaling molecule between neurons [58,59]. As an angiotensin type 1 receptor-related ligand (APJ), the APE-13 system is involved in many physiological as well as pathological processes, such as vasculopathy, energy metabolism, and maintenance of humoral homeostasis and is widely expressed in many tissues and organs, such as the central nervous system (CNS), kidney and other peripheral organs [27,28,60]. 

The demonstrated correlation of APE-13 with the level of eating disorder psychopathology is evidence for a central and multidimensional effect of APE. Regarding the fact that APE-13 expression has been demonstrated in many CNS regions such as the hypothalamus, hippocampus, pituitary gland, spinal cord, cerebellum, corpus callosum, black matter, dorsal sutural nucleus, amygdala, caudate nucleus, and suprachiasmatic and paraventricular nuclei we believe that APE-13 plays a neuroregulatory role not only in memory processes, mood regulation but also eating behaviors [61,62,63]. The regulation of feeding behavior by APE-13, the close relationship between feeding behavior and emotional behavior, and the changes in its levels in patients with depression and psychotic episodes seem to support this thesis [64,65,66,67,68,69].

## 5. Conclusions

Contrary to our original assumptions, APE-13 was higher in malnourished adolescent girls with AN (AN1) than in the post-realimentation (AN2) and CG groups. APE-13 levels appeared to be independent of insulin or glucose levels while correlating with the severity of eating disorder psychopathology, highlighting the need for further efforts to understand circulating neurohormones and neuropeptides and their impact on metabolic health. In addition, the present study showed that plasma ASP levels increased with increasing body weight in AN patients while correlating with the severity of eating disorder symptoms in extreme emaciation, providing evidence that ASP expression pattern is an essential determinant of metabolic health

## 6. Limitations

The present study’s authors are aware of its limitations and cite the most important ones. When interpreting the results of the study, it is essential to remember that peripheral peptide levels do not necessarily reflect their central activity. Recent reports show a possible effect of APE-13 on fertility, which, due to the lack of assessment of sex hormone levels in female patients, was not included in the above study. It also appears to be a methodological limitation of our experiment that the leading manufacturers of APE-13 assay kits have so far not conducted accurate specificity tests to fully exclude contamination with other isoforms, such as [Pyr1]APE-13.

Furthermore, AN’s etiology is complex, and only single biological factors were analyzed. It is difficult to determine which of the changes in protein levels are primary and which are secondary to changes in body weight and severity of psychopathological symptoms. Similarly, this study did not analyze the dynamics of changes in psychopathological symptoms, such as the severity of depressive symptoms over time and their relationship to the rate of weight gain, biochemical or socio-demographic indices. Therefore, following up the studies on a larger group of subjects differing in diagnosis, gender, and BMI is strongly recommended.

## Figures and Tables

**Table 1:** Age, height, body weight, body mass index (BMI), percentage of ideal body mass (% IBW), and fasting plasma levels of glucose, insulin, HOMA-IR, asprosin (ASP) and apelin-13 (APE-13) in adolescents with anorexia nervosa in extremely low body weight (AN1), after partial normalization of body weight (AN2), as well as in the healthy control group (CG).

	Group M ± SD/Me (Q1; Q3)			AN1 vs. AN2		AN1 vs. CG		AN2 vs. CG	
	An1 *n* = 44	An2 *n* = 44	Control *n* = 29	MD (95% CI)	*p*	MD (95% CI)	*p*	MD (95% CI)	*p*
Age	15.50 (14.00; 17.00)		15.00 (14.00; 17.00)	-	-	MD (95% CI) = 0.50 (−1.00; 1.00); *p* = 0.995			
Height (m)	1.61 ± 0.08		1.65 ± 0.05	-	-	MD (95% CI) = −0.04 (−0.06; <0.01); *p* = 0.024 ^3^			
Body weight (kg)	37.00 (32.75; 40.00) 36.82 ± 4.96	45.00 (42.00; 48.25) 44.92 ± 5.81	53.70 (47.00; 56.70)	−8.10 (−10.35; −5.86)	<0.001 ^1^	−16.70 (−18.80; −12.10)	<0.001	−8.70 (−11.00; −4.00)	<0.001
BMI	14.13 (13.55; 15.00) 14.20 ± 1.28	17.08 (15.81; 18.80) 17.48 ± 2.78	18.91 (17.10; 20.98)	−3.28 (−4.23; −2.34)	<0.001 ^1^	−4.78 (−6.29; −3.67)	<0.001	−1.83 (−3.34; −0.55)	0.006
IBW	55.41 ± 3.86		57.26 ± 2.37	-	-	MD (95% CI) = −1.85 (−3.00; <0.01); *p* = 0.024 ^3^			
%IBW	66.39 (62.06; 70.44) 66.30 ± 6.32	78.97 (74.09; 87.80) 81.44 ± 11.77	90.76 (82.64; 98.43)	−15.14 (−19.43; −10.84)	<0.001 ^1^	−24.37 (−30.45; −18.85)	<0.001	−11.79 (−16.88; −3.98)	0.003
ASP [ng/mL]	8.06 (2.98; 13.87)	10.08 (5.64; 20.86)	5.61 (3.23; 9.08)	−2.04 (−7.94; −0.92)	0.008 ^2^	2.45 (−2.83; 4.66)	0.961	4.47 (−0.40; 7.75)	0.075
APE-13 [pg/mL]	113.56 (32.62; 253.56)	50.93 (30.60; 202.18)	68.08 (17.54; 96.94)	30.99 (2.78; 126.76)	0.037 ^2^	45.48 (1.23; 133.96)	0.046	−17.15 (−25.51; 78.08)	0.479
Insulin	5.50 (4.30; 6.65)	10.10 (7.50; 13.50)	8.55 (5.73; 10.13)	−5.20 (−6.95; −2.85)	<0.001 ^2^	−3.05 (−3.90; −0.40)	0.017	1.55 (−0.10; 4.60)	0.069
Glucose	78.00 (74.00; 83.25) 78.02 ± 7.77	79.00 (75.50; 86.50)	77.00 (71.25; 82.00) 77.38 ± 7.53	−1.00 (−9.00; 0.50)	0.075 ^2^	−1.00 (−3.14; 4.42)	0.736 ^3^	2.00 (−1.00; 8.00)	0.163
HOMA-IR	1.08 (0.80; 1.31)	2.23 (1.51; 2.81)	1.52 (1.09; 2.07)	−1.18 (−1.52; −0.64)	<0.001 ^2^	−0.44 (−0.78; −0.09)	0.013	0.71 (0.09; 1.02)	0.026

Data presented as mean ± SD (standard deviation) or median (Q1—quartile 1; Q3—quartile 3), depending on normality of distribution (both were shown if distribution was not normal only in one of the compared groups). MD—mean difference or median difference between groups with 95% confidence intervals. Dependent comparisons were made with Student’s *t*-test for dependent samples (paired *t*-test) ^1^ or Wilcoxon’s test ^2^. Independent comparisons were made using Student’s *t*-test for independent samples ^3^ or Mann–Whitney’s *U* test. In the case of dependent comparisons, the means/medians were given for the whole groups, and the difference in the means/medians for the groups after excluding subjects who did not participate in both measurements of the analyzed variable.

**Table 2:** The Beck Depression Inventory (BDI), the Hamilton Depression Rating Scale (HAMD), Children’s Yale–Brown Obsessive Compulsive Scale (CY-BOCS) and the Eating Attitude Test (EAT-26) scores in adolescents with anorexia nervosa in extremely low body weight (AN1), after partial normalization of body weight (AN2), as well as in the healthy control group (CG).

	Group M ± SD/Me (Q1; Q3)			AN1 vs. AN2		AN1 vs. CG		AN2 vs. CG	
TEST	An1 *n* = 44	An2 *n* = 44	Control *n* = 29	MD (95% CI)	*p*	MD (95% CI)	*p*	MD (95% CI)	*p*
BDI	13.00 (7.25; 24.00)	11.50 (4.00; 22.50)	5.00 (1.00; 11.00)	3.00 (0.50; 6.50)	0.012 ^1^	8.00 (3.00; 15.00)	0.003	6.50 (<0.01; 10.00)	0.042
HAMD	12.00 (8.50; 17.00)	8.00 (2.00; 16.00)	0.00 (0.00; 3.00)	4.50 (0.00; 7.50)	0.050 ^1^	12.00 (8.00; 13.00)	<0.001	8.00 (3.00; 11.00)	<0.001
CYBOCS	8.00 (4.00; 14.00)	2.00 (1.00; 6.00)	2.00 (0.00; 5.00)	6.00 (2.00; 6.00)	<0.001 ^1^	6.00 (1.00; 8.00)	0.006	0.00 (−1.00; 0.00)	0.079
EAT-26	22.00 (15.00; 36.00)	7.00 (3.00; 19.50)	4.50 (2.75; 8.50)	11.00 (6.00; 15.50)	<0.001 ^1^	17.50 (10.00; 22.00)	<0.001	2.50 (<0.01; 10.00)	0.079

Data presented as median (Q1—quartile 1; Q3—quartile 3) due to lack of normality of distribution. MD—median difference between groups with 95% confidence intervals. ^1^ Independent comparisons were made using Mann–Whitney’s *U* test. Dependent comparisons were made using Wilcoxon’s test. In case of dependent comparisons, the medians were given for the whole groups and the difference in the medians for the groups after excluding subjects who did not participate in both measurements of the analyzed variable.

**Table 3:** Correlations between asprosin (ASP) and apelin-13 (APE-13) and metabolic variables: height, body weight, BMI, %IBW, fasting plasma glucose, insulin levels and HOMA-IR in adolescents in treatment for anorexia (AN1, AN2) and in healthy controls (CG).

	AN1				AN2				CG			
	ASP		APE-13		ASP		APE-13		ASP		APE-13	
	rho	*p*	rho	*p*	rho	*p*	rho	*P*	rho	*p*	rho	*p*
Height	−0.32	0.0497	0.16	0.355	−0.50	0.001	0.43	0.008	0.03	0.887	−0.02	0.935
Body weight	−0.35	0.032	0.03	0.856	0.04	0.818	0.20	0.234	0.17	0.394	−0.28	0.286
BMI	−0.26	0.113	−0.06	0.722	0.34	0.028	−0.07	0.660	0.14	0.496	−0.21	0.441
IBW	−0.32	0.0497	0.16	0.355	−0.50	0.001	0.43	0.008	0.03	0.887	−0.02	0.935
%IBW	−0.30	0.070	−0.05	0.779	0.22	0.160	0.04	0.824	0.15	0.470	−0.23	0.389
Glucose	−0.23	0.175	−0.03	0.858	−0.33	0.037	−0.09	0.616	−0.12	0.588	−0.21	0.458
Insulin	−0.11	0.533	−0.19	0.279	−0.43	0.005	0.31	0.068	0.25	0.229	−0.28	0.314
HOMA-IR	−0.15	0.373	−0.09	0.579	−0.49	0.002	0.29	0.087	0.22	0.311	0.27	0.334

rho—Pearson’s correlation coefficient.

**Table 4:** Correlations between asprosin (ASP) and apelin-13 (APE-13) and psychopathological symptoms measured with the Beck Depression Inventory (BDI), the Hamilton Depression Rating Scale (HAMD), Children’s Yale–Brown Obsessive Compulsive Scale (CY-BOCS) and the Eating Attitude Test (EAT-26) scores in adolescents in treatment for anorexia (AN1, AN2) and in healthy controls (CG).

	AN1				AN2				CG			
	ASP		APE-13		ASP		APE-13		ASP		APE-13	
	rho	*p*	rho	*p*	rho	*p*	rho	*p*	rho	*p*	rho	*p*
BDI	0.23	0.298	−0.46	0.034	0.06	0.771	0.28	0.181	−0.26	0.201	−0.09	0.736
HAMD	0.10	0.661	−0.42	0.051	0.06	0.751	0.11	0.579	−0.25	0.214	−0.31	0.242
CYBOCS	0.23	0.291	<0.01	0.998	0.04	0.832	0.15	0.461	−0.21	0.315	−0.16	0.542
EAT-26	0.51	0.025	−0.50	0.028	−0.13	0.588	0.53	0.030	0.19	0.420	0.34	0.237

rho—Pearson’s correlation coefficient.

## Data Availability

Not applicable.

## Abbreviations

AN	anorexia nervosa
AN1	adolescents with anorexia nervosa in extremely low body weight
AN2	adolescents after partial normalization of body weight
APE	apelin
APE-13	apelin-13
ASP	asprosin
BDI	The Beck Depression Inventory
CG	healthy control group
CNS	Central Nervous System
CYBOCS	Children’s Yale–Brown Obsessive Compulsive Scale
EAT-26	the Eating Attitude Test
HAMD	the Hamilton Depression Rating Scale
OCD	Obsessive compulsive disorder
